# Rethinking neglected tropical diseases: A shift towards more inclusive and equitable terminology

**DOI:** 10.1371/journal.pgph.0004094

**Published:** 2025-02-03

**Authors:** Marlous L. Grijsen, Yonah E. Yangaza, Al Kadri, Fidel Strub, Esther E. Freeman, Wendemagegn Enbiale

**Affiliations:** 1 Oxford University Clinical Research Unit Indonesia, Faculty of Medicine Universitas Indonesia, Jakarta, Indonesia; 2 Nuffield Department of Medicine, Centre for Tropical Medicine and Global Health, University of Oxford, Oxford, United Kingdom; 3 One Health Society, Dar es salaam, Tanzania; 4 PerMaTa, Makassar, Indonesia; 5 Elysium Noma Survivors Association, Basel, Switzerland; 6 Department of Dermatology, Massachusetts General Hospital, Boston, Massachusetts, United States of America; 7 Medical Practice Evaluation Foundation, Massachusetts General Hospital, Boston, Massachusetts, United States of America; 8 International Foundation for Dermatology, London, United Kingdom; 9 Department of Dermatology, College of Health Sciences and Medicine, Bahir Dar University, Bahir Dar, Ethiopia; 10 Collaborative Research and Training Center for Neglected Tropical Diseases, Arba Minch University, Arba Minch, Ethiopia; PLOS: Public Library of Science, UNITED STATES OF AMERICA

## Abstract

Neglected tropical diseases (NTD) refer to a group of 21 diseases that disproportionally affect impoverished communities in low- and middle-income countries (LMIC) [1]. NTD collectively impact 1.7 billion people, which is about 20% of the world’s population [1]. Each year, NTD account for more than 200,000 deaths, with millions left disabled and disfigured due to insufficient access to care and affordable treatment, often leading to social exclusion, stigmatization and discrimination. Although the term NTD has successfully directed funding and resources towards these conditions and encouraged global partnerships and high-level policy initiatives, the term may also have unintended negative consequences. In this paper, we aim to explore the term NTD and stimulate a dialogue that re-evaluates its meaning into more inclusive and equitable language.

Since the 1970s, there has been a growing interest in developing a concerted global response to NTD. This led to the establishment of the WHO NTD-department in 2005, aimed at improving the health and well-being of individuals affected by NTD through coordinated global initiatives, mobilization of funds and the implementation of evidence-based interventions [2]. In 2012, the London Declaration of NTD was launched, uniting governments, pharmaceutical companies, and non-governmental organizations (NGO’s) in the fight against NTD. The global response was revitalized with the Kigali Declaration on NTD in 2022, which reaffirmed a collective commitment to adhere to the WHO’s 2021-2030 roadmap for NTD control. Significant milestones have been achieved, including a 25% reduction in the number of people requiring interventions, the elimination of at least one NTD in 47 countries, and a substantial drop in the disease burden calculated in disability-adjusted life years [3]. Moreover, in December 2023, noma, one of the world’s most underrecognized conditions, was designated by WHO as an NTD.

While recognizing that significant progress has been made, we here explore the potential negative consequences associated with the term NTD. First, the word ‘tropical’ dates back to the 12^th^ century and has historical roots tied to colonial history as European powers established colonies in ‘tropical’ regions encountering ‘exotic’ diseases that were prevalent in those settings. ‘Tropical medicine’ emerged in the 19^th^ century and was designed to reinforce colonialism as it was essential to study prevalent diseases that were threatening the white colonizers as mortality rates were high [4]. As the term ‘tropical’ is linked to notions of otherness, exoticism and superiority by colonial powers, this association may instinctively contribute to the perception of NTD as geographically distant and less important, reinforcing a division between the Global North and South. This dynamic reflects deep-rooted power structures, racial biases and accentuates existing health disparities within the field of global health [5].

Second, ‘tropical’ suggests that neglected diseases are confined to geographic areas with a tropical climate, disregarding the reality that some of these diseases are prevalent in or spreading to temperate zones due to shifting epidemiological patterns associated with human behaviour and climate injustice [6]. Examples are scabies, which is endemic across the world, including the Global North, and the emergence of dengue and leishmaniasis in France [7,8], chikungunya in Italy [9] and Chagas disease in Spain [10].

Third, labelling diseases as ‘neglected’ carries a negative perception of communities that often already are marginalized and stigmatized. “When I was diagnosed with leprosy and read it was considered an NTD, I was surprised as so many people in my neighbourhood were affected by this condition.” [*Al Kadri, chair of PerMaTa, an independent locally driven leprosy association by and for persons affected by leprosy in Indonesia*]. Fidel Strub, a noma survivor, advocate, and co-founder and director of Elysium Noma Survivors Association, challenges the use of the term ‘neglected’ due to its controversial nature. He argues that while the term may have significance at a global level, it may also downplay the severity of the issue and imply that only a small percentage of individuals are impacted. Strub believes that labelling the issue as ‘neglected’ diminishes the urgency for change and may not convince governments and donors to invest more. Pejorative language may perpetuate stereotypes and contribute to stigma and discrimination [11]. It is important to understand the meaning of words and the influence language has on shaping attitudes. A powerful example comes from Ethiopia where NTD is translated into ‘tropical diseases needing special attention’ underlining the need for action [12].

Fourth, the term NTD fails to acknowledge the human beings impacted by these conditions. NTD have long existed within communities, yet individuals affected by them have often been ignored. Mathias Duck, a global health advocate and a person with lived experience of leprosy, introduced the term ‘diseases of neglected people’ [13]. He proposes to shift from a disease-centric perspective to one that centres persons affected: listening to their experiences, addressing their needs and involving them in decision-making procedures.

Finally, the word ‘neglected’ may unintentionally signal lack of importance to local governments, potentially leading to less funding and political determination. This perception may reinforce a passive acceptance of the situation, diminish country ownership and encourage reliance on external donors rather than fostering change [12]. “NTD should be seen as a powerful expression, but because the diseases are neglected, our government thinks they are no longer important” [*Al Kadri*]. While the NTD-terminology may convey a sense of urgency and prompt advocacy, it can also paradoxically impede access to essential treatments, preventive measures and innovative research and prolong suffering and avertible disability among affected populations.

The introduction of the term NTD has played a key role in raising awareness, mobilizing political willpower, and securing funding. However, it is time to contemplate the potential sensitivities associated with the terminology and embrace more inclusive person-centred language transcending social and geographical boundaries.

We acknowledge the way forward is complex, with no single term currently identified as ideal. However, several precedents offer valuable insights, i.e., the renaming of problematic medical eponyms [14] and the emergence of race-conscious medicine [15]. Several schools or departments of tropical medicine have been renamed as schools or institutes of international or global health. Other examples are the shift to ‘person living with HIV’ or ‘person with albinism’, which exemplifies how language can be restructured to prioritize the individual over the medical condition and how community consensus can drive change. To improve the depth of understanding related to NTD, stakeholder consultations and community engagement may help to capture a diverse range of perspectives from individuals affected, the general public, healthcare professionals, NGO’s, local governments and policymakers. Inspired by the Ethiopian example, we propose the term ‘conditions requiring special attention’, which emphasizes the global nature, the focus on achieving health justice for all individuals affected and the need for collective action. Although this term may have limitations, our primary goal is to amplify the voices of those affected and promote dialogue within the global health community to generate alternative suggestions that are more inclusive and respectful of all perspectives.
